# TLR–Dependent Control of *Francisella tularensis* Infection and Host Inflammatory Responses

**DOI:** 10.1371/journal.pone.0007920

**Published:** 2009-11-20

**Authors:** Allison L. Abplanalp, Ian R. Morris, Bijaya K. Parida, Judy M. Teale, Michael T. Berton

**Affiliations:** 1 Department of Microbiology and Immunology, University of Texas Health Science Center at San Antonio, San Antonio, Texas, United States of America; 2 Department of Biology, University of Texas at San Antonio, San Antonio, Texas, United States of America; University of Toronto, Canada

## Abstract

**Background:**

*Francisella tularensis* is the causative agent of tularemia and is classified as a Category A select agent. Recent studies have implicated TLR2 as a critical element in the host protective response to *F. tularensis* infection, but questions remain about whether TLR2 signaling dominates the response in all circumstances and with all species of *Francisella* and whether *F. tularensis* PAMPs are predominantly recognized by TLR2/TLR1 or TLR2/TLR6. To address these questions, we have explored the role of Toll-like receptors (TLRs) in the host response to infections with *F. tularensis* Live Vaccine Strain (LVS) and *F. tularensis* subspecies (subsp.) *novicida in vivo*.

**Methodology/Principal Findings:**

C57BL/6 (B6) control mice and TLR– or MyD88-deficient mice were infected intranasally (i.n.) or intradermally (i.d.) with *F. tularensis* LVS or with *F. tularensis* subsp. *novicida*. B6 mice survived >21 days following infection with LVS by both routes and survival of TLR1^−/−^, TLR4^−/−^, and TLR6^−/−^ mice infected i.n. with LVS was equivalent to controls. Survival of TLR2^−/−^ and MyD88^−/−^ mice, however, was significantly reduced compared to B6 mice, regardless of the route of infection or the subspecies of *F. tularensis*. TLR2^−/−^ and MyD88^−/−^ mice also showed increased bacterial burdens in lungs, liver, and spleen compared to controls following i.n. infection. Primary macrophages from MyD88^−/−^ and TLR2^−/−^ mice were significantly impaired in the ability to secrete TNF and other pro-inflammatory cytokines upon *ex vivo* infection with LVS. TNF expression was also impaired *in vivo* as demonstrated by analysis of bronchoalveolar lavage fluid and by *in situ* immunofluorescent staining.

**Conclusions/Significance:**

We conclude from these studies that TLR2 and MyD88, but not TLR4, play critical roles in the innate immune response to *F. tularensis* infection regardless of the route of infection or the subspecies. Moreover, signaling through TLR2 does not depend exclusively on TLR1 or TLR6 during *F. tularensis* LVS infection.

## Introduction

*Francisella tularensis* is a Gram-negative, coccobacillus that replicates within macrophages, neutrophils, hepatocytes and type II lung epithelial cells [1]–[5], and causes the zoonotic disease tularemia in mammalian hosts [6]. Infection of humans occurs naturally by exposure to infected animal carcasses, insect bites, ingestion or inhalation. There are four subspecies (subsp.): *tularensis* (type A), *holarctica* (type B), *mediasiatica*, and *novicida*. Subsp. *tularensis* strains are highly virulent (LD_50_<10–100 CFU) [7], [8], and cause severe disease and death sporadically, predominantly in North America. Subsp. *holarctica* strains cause a more widespread but less severe disease in Northern Europe, Scandinavia and the former Soviet Union. Subsp. *novicida* is highly attenuated for humans, and is rarely isolated, but causes lethal disease in mice [9], [10]. Because of the high infectivity, virulence, and ability to be disseminated by aerosol, *F. tularensis* type A and B strains have been classified as Category A bioweapon agents [11]. A live attenuated vaccine strain (derived from a subsp. *holarctica* strain and known as LVS) was developed 50–60 years ago [12], but a lack of knowledge about the mechanisms of attenuation and concerns about reversion to virulence have prevented its licensure for use in the U.S. [6], [11]. Although LVS is attenuated in humans, it causes a disease in mice that is very similar to human tularemia, and thus it has been used extensively to model the human disease [13], [14].

A hallmark of *F. tularensis* is its ability to infect, replicate and survive within many cell types, including macrophages and neutrophils (reviewed in [15]). The mechanisms used by *F. tularensis* to evade host cellular defenses remain largely unknown but appear to involve the abilities to escape the phagosome and replicate in the cytoplasm [16]–[18], block the respiratory burst in neutrophils [4], and suppress or delay inflammatory cytokine production [2], [19], [20]. Most of what is known about the host immune response to *F. tularensis* has come from studies in mice infected intradermally (LD_50_ of ∼10^6^) or intraperitoneally (LD_50_<10) with LVS [13], [21], although recent studies have begun to focus on pulmonary infection [22]–[24]. The emerging picture is that the immune response to infection with *F. tularensis* involves IFN-γ- and TNF-mediated activation of resident macrophages and recruited neutrophils that are important for controlling the initial infection, and CD4^+^ and CD8^+^ T cells that are required to fully resolve the infection and produce long-term protective immunity (reviewed in [14]). Recent evidence also supports a role for the ASC/caspase-1/IL-1 axis in this infection [25]. The mechanisms by which *F. tularensis* evades the host innate immune response and rapidly replicates and disseminates to other organs to establish systemic infection are unknown.

Innate immune responses are initiated as a result of recognition by pattern recognition receptors (PRRs) of conserved molecules expressed by many pathogens (pathogen-associated molecular patterns or PAMPs). The Toll-like receptors (TLRs) are evolutionarily conserved, germline-encoded PRRs that signal many different cell types via a set of conserved signaling adaptors/molecules that also participate in IL-1 receptor signaling (reviewed in [26]). TLR signaling primarily activates the NF-κB and MAPK signaling pathways, both of which play important roles in inflammatory responses. The cytoplasmic domains of the TLRs share a conserved domain with the IL-1 receptor known as the Toll/IL-1 receptor (TIR) domain. This domain recruits other TIR domain-containing adapter proteins, such as MyD88, that mediate downstream signaling to activate pro-inflammatory gene expression. Certain TLRs form complexes with other accessory molecules or form heterodimers with other TLRs. For example, TLR2 dimerizes with either TLR1 or TLR6 to recognize triacylated lipopeptides or diacylated lipopeptides, respectively [27]–[30]. The PRRs that recognize *F. tularensis* PAMPs *in vivo* are just beginning to be identified [31]. Recent *in vivo* studies have indicated that TLR2 is critical for protection against intranasal *F. tularensis* infection but not against intradermal infection [32], [33]. Our studies demonstrate that TLR2 signaling via MyD88 plays an important role in innate immune responses to *F. tularensis* infection *in vivo* regardless of the route of infection and regardless of the subspecies of *F. tularensis*. We also demonstrate that neither TLR1 nor TLR6 is exclusively required for TLR2-dependent recognition of *F. tularensis in vivo*.

## Materials and Methods

### Ethics statement

All animals were handled in strict accordance with good animal practice, the Animal Welfare Act, the U.S. Public Health Service Policy on Humane Care and Use of Laboratory Animals, and “Guide for the Care and Use of Laboratory Animals” published by the National Research Council. All animal work was approved by the University of Texas Health Science Center at San Antonio Institutional Animal Care and Use Committee (protocol # 03062A-34-04-A,C).

### Bacteria

The *F. tularensis* Live Vaccine Strain (LVS) (ATCC 29684), originally derived from the fully virulent *F. tularensis* subsp. *holarctica* [12], was provided by Dr. K. Elkins (Center for Biologics Research and Evaluation, U.S. Food and Drug Administration, Bethesda, MD). *F. tularensis* subsp. *novicida* strain U112, originally isolated from a water sample in Utah [9], [34], was obtained from Dr. Karl Klose (University of Texas at San Antonio, San Antonio, TX). LVS was grown at 37°C in tryptic soy broth supplemented with 1% IsoVitaleX (BD Diagnostic Systems, Sparks, MD) and subsp. *novicida* was grown in the same medium plus 0.1% L-cystine. Frozen stocks were stored in 40% glycerol at −80°C. Inocula for infections of mice were prepared by first streaking a glycerol bacterial stock on chocolate agar. After 72 h, bacterial colonies were harvested and spread on chocolate agar to form a lawn. After 24 h, the lawn of bacterial growth was scraped from the plate and thoroughly dispersed in tryptic soy broth containing the appropriate supplements described above. Bacterial inocula prepared in this way were stored at 4°C and used within 1 week of preparation, during which the titer of viable bacteria was stable. The titer of the inoculum used in a particular mouse infection was determined by plating serial dilutions on chocolate agar plates on the day of infection. All protocols were approved by the Institutional Biosafety Committee at the University of Texas Health Science Center at San Antonio.

### Mice

C57BL/6 (B6) mice were obtained from the National Cancer Institute (NCI-Frederick Animal Production Area, Frederick, MD). B6129PF2/J hybrid mice (C57BL/6×129P) were purchased from The Jackson Laboratory (Bar Harbor, ME). TLR1^−/−^ (N4) [30], TLR2^−/−^ (N4) [35], TLR4^−/−^ (N4) [36], TLR6^−/−^ (N5) [37] and MyD88^−/−^ (N4) [38] breeding pairs (all backcrossed for 4–5 generations onto the C57BL/6 genetic background) were obtained under a materials transfer agreement from Dr. Shizuo Akira (Osaka University, Osaka, Japan) via Dr. Douglas Golenbock (University of Massachusetts Medical School, Worcester, Mass.). Mice were used at 8–16 weeks of age in these studies. Mice were bred and maintained in ventilated cages under specific pathogen-free conditions in the University of Texas Health Science Center at San Antonio Laboratory Animal Resources Department, an AAALAC-accredited facility.

### Mouse infection and determination of bacterial burden

Bacterial inoculum stocks were diluted in phosphate-buffered saline (PBS) to the appropriate CFU/ml. Mice were anesthetized lightly by intramuscular injection of a ketamine cocktail (30 mg/ml ketamine, 4 mg/ml xylazine), and infected intranasally, or intradermally at the base of the tail [21], with 20 µl of inoculum. The serially diluted inoculum was immediately plated on chocolate agar to determine the actual CFU/ml delivered for each experiment. To measure bacterial burden, mice were sacrificed at 1, 3, 5 and 7 days after intranasal (i.n.) infection, and the lungs, left lobe of liver and spleen were removed aseptically and homogenized in 5 ml of PBS. Ten-fold serial dilutions were prepared and plated on chocolate agar and the number of CFU in each dilution was determined after 72 h of incubation at 37°C.

### Mouse survival studies

Groups of mice were infected with *F. tularensis* LVS or subsp. *novicida* as described above and monitored twice daily for 21 days for signs of illness and death. Kaplan-Meier product-limit analysis and Log Rank analysis of survival data was performed with SigmaStat 3.1 software (Systat Software, Inc., Point Richmond, CA). The Holm-Sidak multiple comparison method was used to compare survival curves from all groups of mice, and a *p* value of <0.05 was considered significant.

### Bronchoalveolar lavage (BAL)

Mice were sacrificed and the tracheas were exposed through midline incision and cannulated with a sterile 18-gauge BD Angiocath™ catheter (Becton Dickinson Infusion Therapy Systems Inc., Sandy, UT). The lungs were lavaged serially with 1-ml aliquots of sterile lavage solution (PBS, 3 mM EDTA, 0.1 mM isoproterenol) for a total of 5 ml. The serial lavage aliquots recovered from a single mouse were pooled (∼4 ml) and mixed with an equal volume of complete RPMI medium (RPMI 1640, 10% FBS, 2% penicillin-streptomycin, 2 mM L-glutamine, 1 mM sodium pyruvate, 100 µM non-essential amino acids, 50 µM β-mercaptoethanol). BAL cells were pelleted and the resulting supernatant (bronchoalveolar lavage fluid or BALF) was stored at −80°C until use. BAL cells were washed twice with ice-cold culture medium, and cell viability and concentration were determined by staining with trypan blue (0.04%).

### Proteose peptone-elicited peritoneal macrophages

Mice were injected intraperitoneally with 1 ml 10% (w/v) proteose peptone (Fluka, BioChemika, Germany) and were sacrificed 72 h later. Peritoneal cells were harvested by peritoneal lavage with complete RPMI medium and seeded at 1×10^6^ cells/ml into tissue culture dishes. Non-adherent cells were removed after 4 h by extensive washing with phosphate-buffered saline (PBS). Adherent cells were incubated in complete RPMI medium at 37°C, 6% CO_2_ for another 24 h before use.

### *In vitro* infections

Peritoneal macrophages or BAL cells were seeded in 96-well culture plates (5×10^4^–1×10^5^ cells/well) and incubated in antibiotic-free medium for 24 h at 37°C, 6% CO_2_ before use. Cells were washed with complete RPMI medium and infected with LVS (MOI of 80–120) or stimulated in parallel with *E. coli* LPS (10 µg/ml; Sigma-Aldrich #L2360) in triplicate in 0.1–0.2 ml of antibiotic-free medium for 4 h at 37°C, 6% CO_2_. Supernatants harvested from infected cells were frozen at −80°C until use.

### Cytokine assays

Cell culture supernatants or BALFs were thawed and assayed using the BD™ Cytometric Bead Array (CBA) Mouse Inflammation kit (BD Biosciences, San Diego, CA) or by ELISA (BD OptEIA^TM^, BD Biosciences, San Diego, CA) according to the manufacturers' protocols. Analysis of sample data was performed with BD™ CBA Software or with SoftMax® Pro 5 (Molecular Devices, Sunnyvale, CA).

### Immunofluorescence microscopy of frozen lung sections

Mice were infected intranasally with LVS and sacrificed at serial time points. After perfusion with ice-cold PBS, separated lungs were embedded in Tissue-Tek OCT compound and kept at −80°C. Lungs were sectioned at 9 µm by using a Shandon Cryotome SME (Thermo Electron Corporation, Pittsburg, PA). One in every five slides containing lung sections were fixed in formalin for 10 min at room temperature (RT) and stained with H&E to determine the state of the lung as well as the degree of cell infiltration. The rest of the slides were air dried overnight and fixed in fresh acetone for 20 sec at RT. Acetone fixed sections were wrapped in aluminum foil and stored at −80°C. Frozen, fixed lung sections were thawed at RT for 30 min, fixed at −20°C in acetone followed by treatment with 70% ethanol and hydration in PBS. Non-specific binding of antibody reagents was minimized by incubating each slide for 30 min at RT with serum from the same species from which the fluorochrome-conjugated antibodies were derived. Lung sections were then incubated for 40 min with primary antibodies diluted in species-specific serum at a concentration that had been optimized previously. Lung sections were washed 7 times for 3 min each and secondary antibodies (when necessary) were applied and incubated for 30 min at RT. Lung sections were analyzed for expression of TNF using purified goat anti-mouse TNF (#AF-410-NA, R&D Systems, Minneapolis, MN) followed by secondary rhodamine red X-conjugated AffiniPure anti-goat IgG (Jackson ImmunoResearch Laboratories, Inc., West Grove, PA), or for the presence of LVS using Alexa 488-conjugated mouse anti-LVS (provided by Dr. John Gunn, Ohio State University Medical School). Stained sections were mounted with fluorsave reagent (Calbiochem, La Jolla, CA) containing 0.3 uM 4′, 6-diamidino-2-phenylindole (DAPI), dilactate (Molecular Probes, Eugene, OR). Fluorescence was visualized with a Leica DMR epifluorescent microscope (Leica Microsystems, Wetzlar Germany). Images were acquired using a cooled CCD SPOT RT camera (Diagnostic Instruments Inc., Sterling Heights, MI), and were processed and analyzed using Adobe Photoshop 7.0 (Adobe Systems, Inc., Mountain View, CA).

### Statistical analysis

All numerical data was presented as the mean+standard error of the mean (SEM) or the median plus the 25^th^ and 75^th^ percentiles. Significance among groups was determined using one-way ANOVA followed by the Holm-Sidak method of post-hoc analysis for normally distributed data. Alternatively, non-parametric analysis was performed with the Kruskal-Wallis ANOVA on ranks followed by Dunn's post-hoc analysis for multiple comparisons. All statistical analyses were performed with SigmaStat3.1 software (San Jose, CA).

## Results

### MyD88^−/−^ and TLR2^−/−^ mice are more susceptible to infection with *F. tularensis* regardless of the route of infection or the subspecies

**Figure 1:**
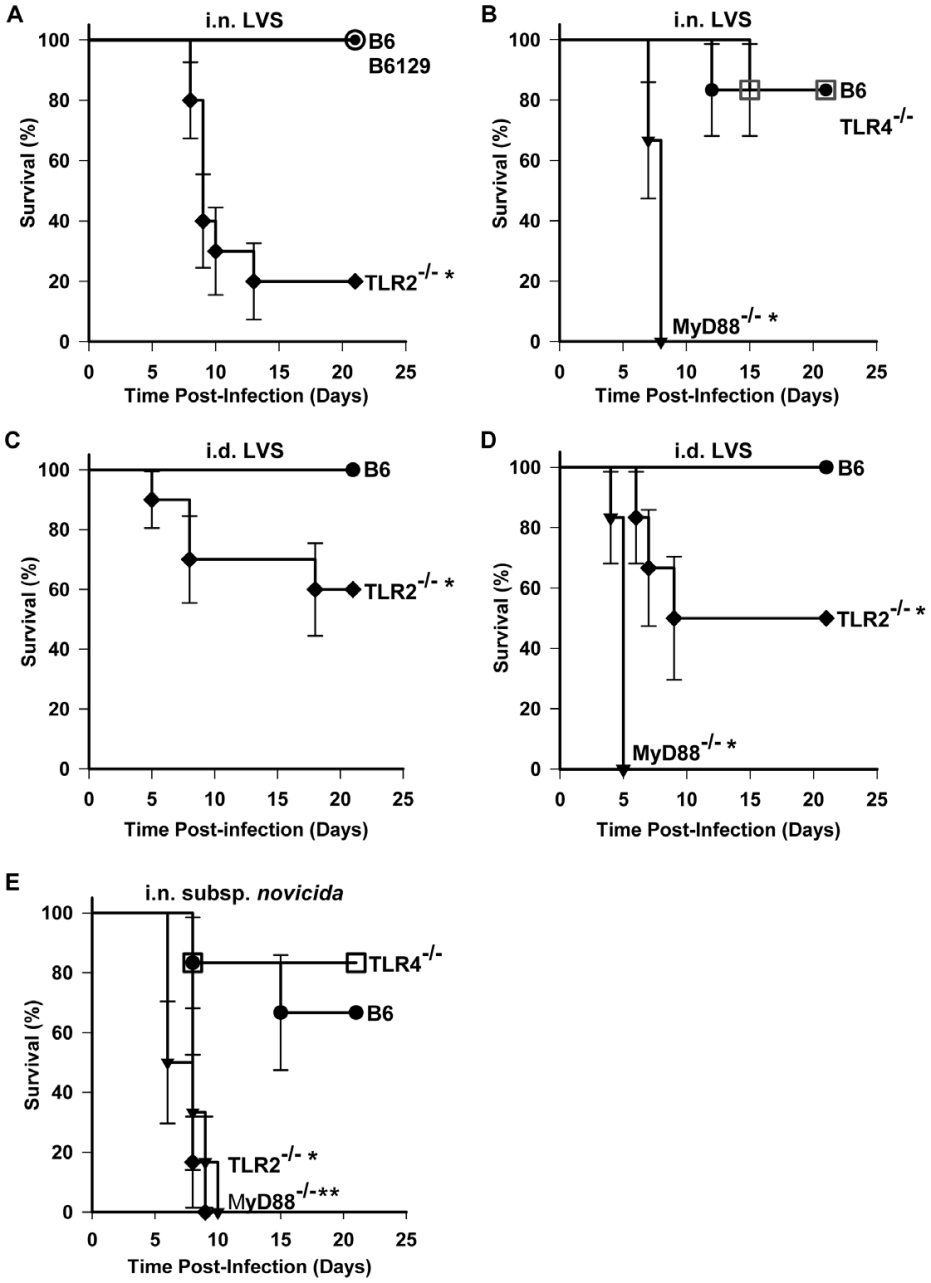
Survival of TLR^−/−^ and MyD88^−/−^ mice inoculated intranasally or intradermally with *F. tularensis*. Groups of mice (•, B6; ○, B6129PF2/J; ♦, TLR2^−/−^; □, TLR4^−/−^; ▾,MyD88^−/−^) were inoculated i.n. or i.d. with the indicated strains. The mice were monitored daily for 21 days for survival and signs of illness. Kaplan-Meier survival analyses were performed and survival was plotted as a function of time with each point representing the cumulative probability of survival for the indicated group. Error bars represent the standard error for the cumulative probability of survival. Significant differences among groups were determined by Log Rank analysis and individual *p* values were calculated by the Holm-Sidek method as described in Materials and Methods; *p* values of <0.05 were considered significant. (A) The i.n. inoculum for B6 and TLR2^−/−^ mice was 4,330 CFU of LVS; i.n. inoculum for B6129PF2/J mice was 6,183 CFU; n = 10. One representative experiment is shown of two independent experiments performed. *p = 0.001. (B) The i.n. inoculum was 5,360 CFU of LVS, n = 6. One representative experiment is shown of 2–4 independent experiments performed. *p = 0.001. (C) The i.d. inoculum was 38,680 CFU of LVS, n = 10. *p = 0.029. (D) The i.d. inoculum was 45,600 CFU, n = 6. *p = 0.055; **p = 0.001. (E) The i.n. inoculum was 4 CFU of *F. tularensis* subsp. *novicida*, n = 6. *p = 0.004; **p = 0.004.

Several recent studies have reported a role for TLR2 in the innate immune response to *F. tularensis* [32], [33], [39], [39]–[42]. Of these, the only two *in vivo* studies, one exploring an intranasal infection model in mouse [32] and the other an intradermal model [33], came to different conclusions regarding the TLR2-dependence of mouse susceptibility to *F. tularensis* LVS infection. We have studied the role of TLRs and TLR signaling pathways in the host response to *F. tularensis* infection *in vivo* following intranasal (i.n.) or intradermal (i.d.) infection with *F. tularensis*. Initially, groups of TLR2^−/−^, TLR4^−/−^ or MyD88^−/−^ mice were infected intranasally with the LVS strain of *F. tularensis* subsp. *holarctica*. Control groups of C57BL/6 (B6) mice and B6129PF2 hybrid mice were also infected in parallel. Mice were observed for 21 days to determine morbidity and mortality. Figure 1A and 1B demonstrate that all but one B6 and all B6129PF2 mice survived when infected with LVS intranasally (<1 LD_50_), although all mice became obviously ill. The clinical course and survival of TLR4^−/−^ mice were indistinguishable from control mice (Figure 1B). TLR2^−/−^ and MyD88^−/−^ mice, however, showed significantly increased susceptibility to i.n. infection (Figure 1A and 1B). Eight of ten TLR2^−/−^ mice died by day 13 post-infection with a median time-to-death (MTD) of 11.7±1.6 days. MyD88^−/−^ mice had a MTD of 7.7±0.2 days. To assess the influence of the route of infection, we performed intradermal (i.d.) inoculations with a sublethal dose of LVS (∼1/40 of the LD_50_ in B6 mice [21]) and monitored the mice for survival. Figure 1C and 1D show that MyD88^−/−^ and TLR2^−/−^ mice infected intradermally also demonstrate significantly reduced survival and increased mortality rates compared to B6 controls, indicating that mouse survival is dependent on TLR2 signaling regardless of the route of infection.

*F. tularensis* subsp. *novicida* is highly virulent in mice (LD_50_ = <10) [43] and also possesses a distinct and more endotoxic LPS than the LVS strain [10]. We therefore tested whether the survival rate of mice infected intranasally with the subsp. *novicida* would be similarly dependent on TLR2 and not dependent on TLR4. Figure 1E demonstrates that TLR2^−/−^ and MyD88^−/−^ mice infected i.n. with 4 CFU of *F. tularensis* subsp. *novicida* were significantly more susceptible to infection (MTDs of 8±1.5 days and 6±2.5 days, respectively) with this subspecies than B6 control mice (MTD of >21 days), but the TLR4^−/−^ mice (MTD of >21 days) are no more susceptible than control mice. Taken together, these data indicate that TLR2 signaling, but not TLR4 signaling, is critical for survival of infection with *F. tularensis*, regardless of subspecies or route of infection. The increased susceptibility of MyD88-deficient mice relative to TLR2^−/−^ mice suggests that additional MyD88-dependent pathways may also play essential roles in the host response to this infection.

### TLR2^−/−^ and MyD88^−/−^ mice are less able to control *F. tularensis* LVS growth and dissemination

**Figure 2:**
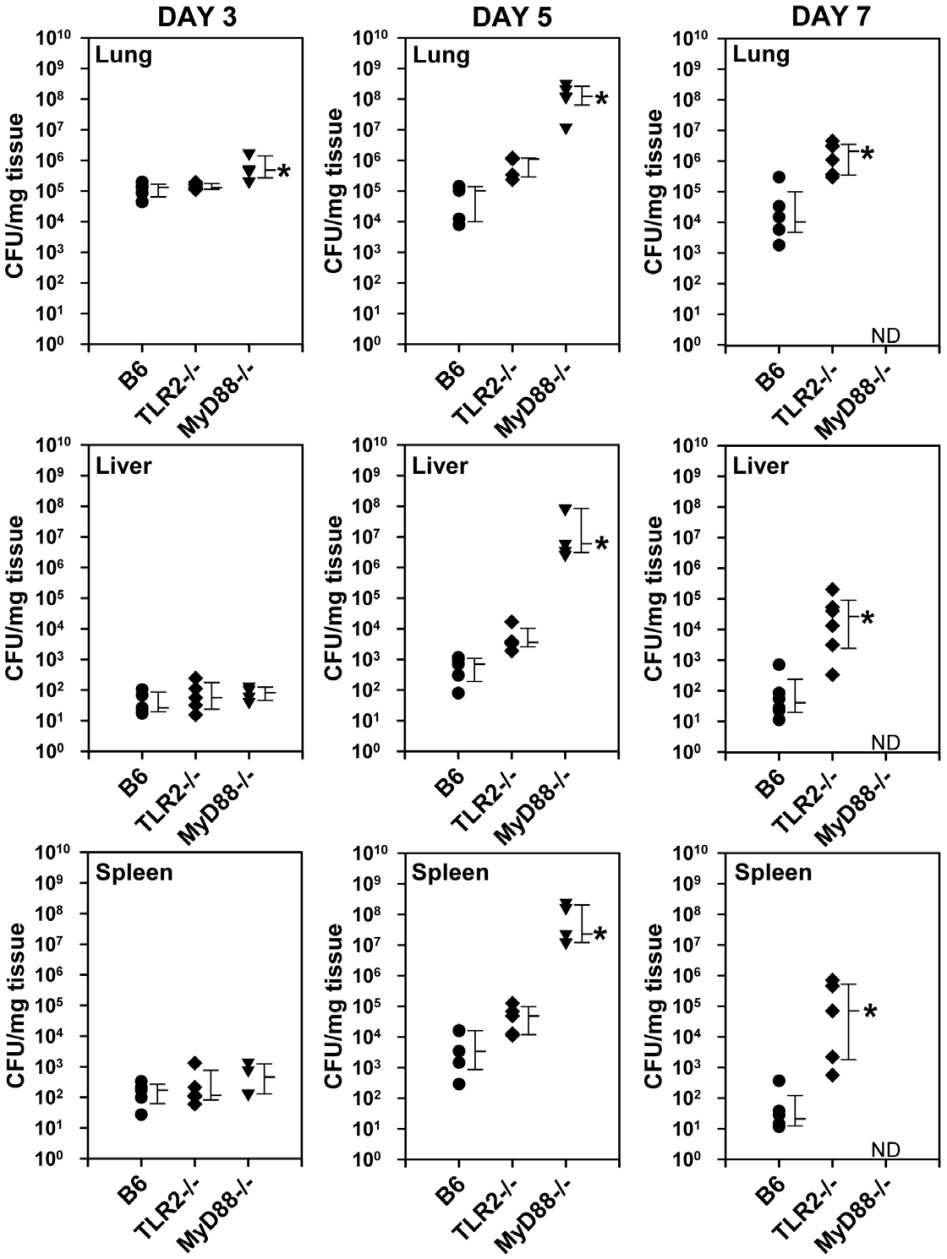
Bacterial burdens in organs of mice following intranasal infection with *F. tularensis* LVS. Groups of mice (•, B6; ♦, TLR2^−/−^; ▾, MyD88^−/−^) were inoculated intranasally with 5000 CFU of LVS and viable bacterial counts were determined in the lungs, livers, and spleens at days 3, 5, and 7 post-infection as described in Materials and Methods. Each data point represents the total CFUs recovered per mg of tissue from the indicated organs from an individual mouse. The median detectable CFU/mg of organ tissue in each group is indicated to the right of the symbols by a horizontal tick mark and the 75^th^ (upper) and 25^th^ (lower) percentiles are also indicated. The data are representative of four independent experiments. Significant differences among groups were determined by Kruskal-Wallis ANOVA on ranks as described in Materials and Methods; n = 5 mice per time-point, *p<0.05 compared to B6 controls.

To determine whether the increased susceptibility of TLR2^−/−^ and MyD88^−/−^ mice to infection with *F. tularensis* was the result of a failure to control bacterial growth and dissemination, the bacterial burden in various organs was determined at different times after intranasal infection with LVS. On days 1, 3, 5 and 7 post-infection, organs were removed from groups of infected mice and viable bacterial counts were determined for the homogenized tissues. No differences among the groups of mice were detected on days 1 (data not shown) and 3 post-infection, except in the lungs of MyD88-deficient mice in which median bacterial burden was modestly higher on day 3 than in lungs of B6 mice (Figure 2). By day 5, the median bacterial burdens in lungs, liver and spleen were modestly higher in TLR2-deficient mice (although not statistically significant) and were 3–4 logs higher in MyD88-deficient mice compared to controls. By day 7, median bacterial burdens in lungs, liver and spleen from TLR2^−/−^ mice were 2–4 logs higher than in organs from controls, but bacterial organ burdens could not be determined for the day 7 post-infection group of MyD88^−/−^mice because the mice all died between days 6 and 7. Consistent with the survival data, the bacterial burdens in the organs of TLR4^−/−^ mice were indistinguishable from those in control mice (data not shown). These results suggest that the increased susceptibility to pulmonary infection with *F. tularensis* in TLR2^−/−^ and MyD88^−/−^ mice results from a failure to limit bacterial replication and dissemination.

### Cytokine and chemokine responses to *F. tularensis* LVS are impaired in macrophages isolated from TLR2^−/−^ and MyD88^−/−^ mice

**Figure 3:**
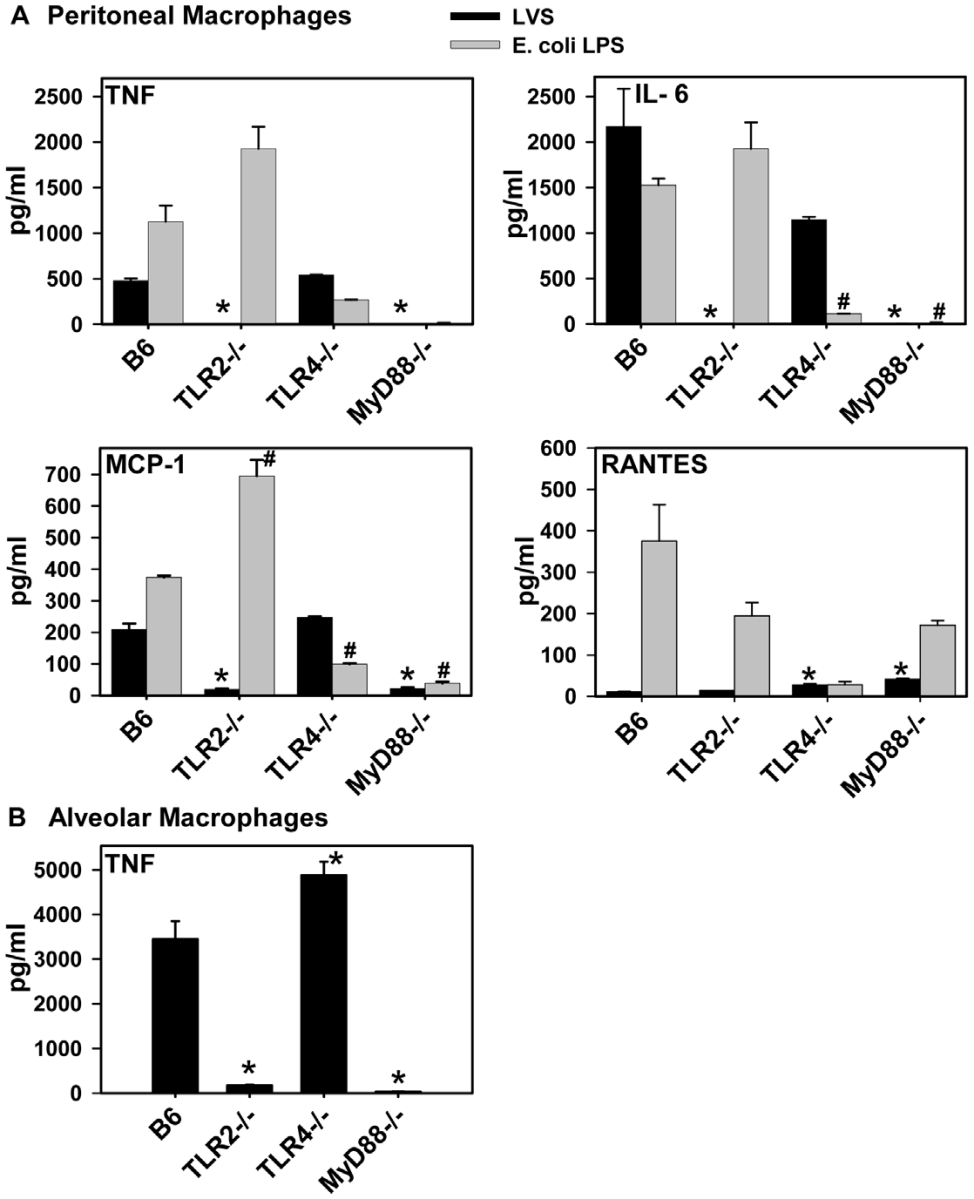
Cytokine and chemokine expression by primary macrophages in response to *F. tularensis* LVS infection. (A) Proteose peptone-elicited peritoneal macrophages from B6, TLR2^−/−^, TLR4^−/−^, and MyD88^−/−^ mice were infected with LVS (MOI of 120) or stimulated with *E. coli* LPS (10 µg/ml) as indicated. Culture supernatants were collected after 4 h, and cytokines were quantified by BD™ Cytometric Bead Array (CBA) Mouse Inflammation Kit (BD Biosciences Pharmingen, San Diego, CA). The data are expressed as the average cytokine level (pg/ml) (+SEM) in duplicate culture supernatants and are representative of 2–3 independent experiments. Significant differences among groups were determined by one-way ANOVA followed by Holm-Sidek post-hoc analysis; ^*^ *p*<0.01 compared to B6 macrophages infected with LVS, ^#^ *p*<0.01 compared to B6 macrophages stimulated with *E. coli* LPS. Cytokine and chemokine levels in supernatants from mock-infected cells were <20 pg/ml (data not shown). (B) Alveolar macrophages harvested by bronchoalveolar lavage were infected with LVS (MOI of 80) and culture supernatants were collected at 4 h. TNF expression was analyzed by ELISA as described in Materials and Methods. The data are expressed as the average TNF level (pg/ml) (+SEM) in duplicate culture supernatants, ^*^ *p*<0.01. TNF levels in supernatants from mock-infected cells were <20 pg/ml (data not shown).

An important immune evasion mechanism for *F. tularensis* is its ability to replicate and survive within macrophages [1]. Macrophages express both TLR2 and TLR4 and make early cytokine and chemokine responses that play a critical role in innate immunity. We therefore investigated the ability of *F. tularensis* to induce cytokines and chemokines from primary mouse macrophages derived from wild-type B6 mice and MyD88^−/−^, TLR2^−/−^, and TLR4^−/−^ mice. As a positive control, macrophages were stimulated in parallel with *E. coli* LPS. Figure 3A (black bars) shows that peritoneal macrophages from B6 mice respond to LVS infection *in vitro* by producing increased amounts of TNF, IL-6 and MCP-1 relative to uninfected cells (<20 pg/ml). No IL-12p70, or IFN-γ were detected in these cultures (data not shown). By contrast, peritoneal macrophages obtained from MyD88^−/−^ mice and TLR2^−/−^ mice demonstrated profoundly impaired TNF, IL-6 and MCP-1 secretion in response to LVS infection compared to macrophages from B6 mice. The failure of TLR2^−/−^ and MyD88^−/−^ macrophages to produce proinflammatory cytokines in response to LVS was not due to a general defect in the cells from these mice because TLR2^−/−^ macrophages secreted significant levels of TNF, IL-6 and MCP-1, and MyD88^−/−^ macrophages secreted a significant level of RANTES, in response to *E. coli* LPS (Figure 3A, gray bars). TNF, IL-6 and MCP-1 responses from TLR4-deficient macrophages infected with LVS were similar to those from wild-type macrophages (Figure 3A, black bars). We also measured TNF secretion by cells obtained from the lungs of mice by bronchoalveolar lavage. BAL cells from MyD88^−/−^ and TLR2^−/−^ mice failed to secrete TNF in response to *in vitro* infection with LVS but cells from B6 and TLR4^−/−^ mice made robust TNF responses *in vitro* (Figure 3B). Similar results were observed in bone marrow-derived macrophages and in macrophages infected with *F. tularensis* subsp. *novicida* (data not shown). Interestingly, TNF, IL-6 and MCP-1 responses to *E. coli* LPS were greater in TLR2-deficient peritoneal macrophages than in control macrophages (Figure 3A, gray bars), and TNF expression in response to LVS infection was greater in TLR4-deficient alveolar macrophages than in B6 controls (Figure 3B). Similar observations have been made by others [44], but why this occurs is not known. It could be the result of increased expression of one TLR in the absence of the other (e.g. increased TLR4 in the absence of TLR2), increased availability of downstream signaling molecules in the TLR-deficient cells, or some other form of cross-regulation between these signaling pathways.

### TLR2^−/−^ and MyD88^−/−^ mice exhibit impaired *in vivo* expression of cytokine and chemokine expression in the lungs

**Figure 4:**
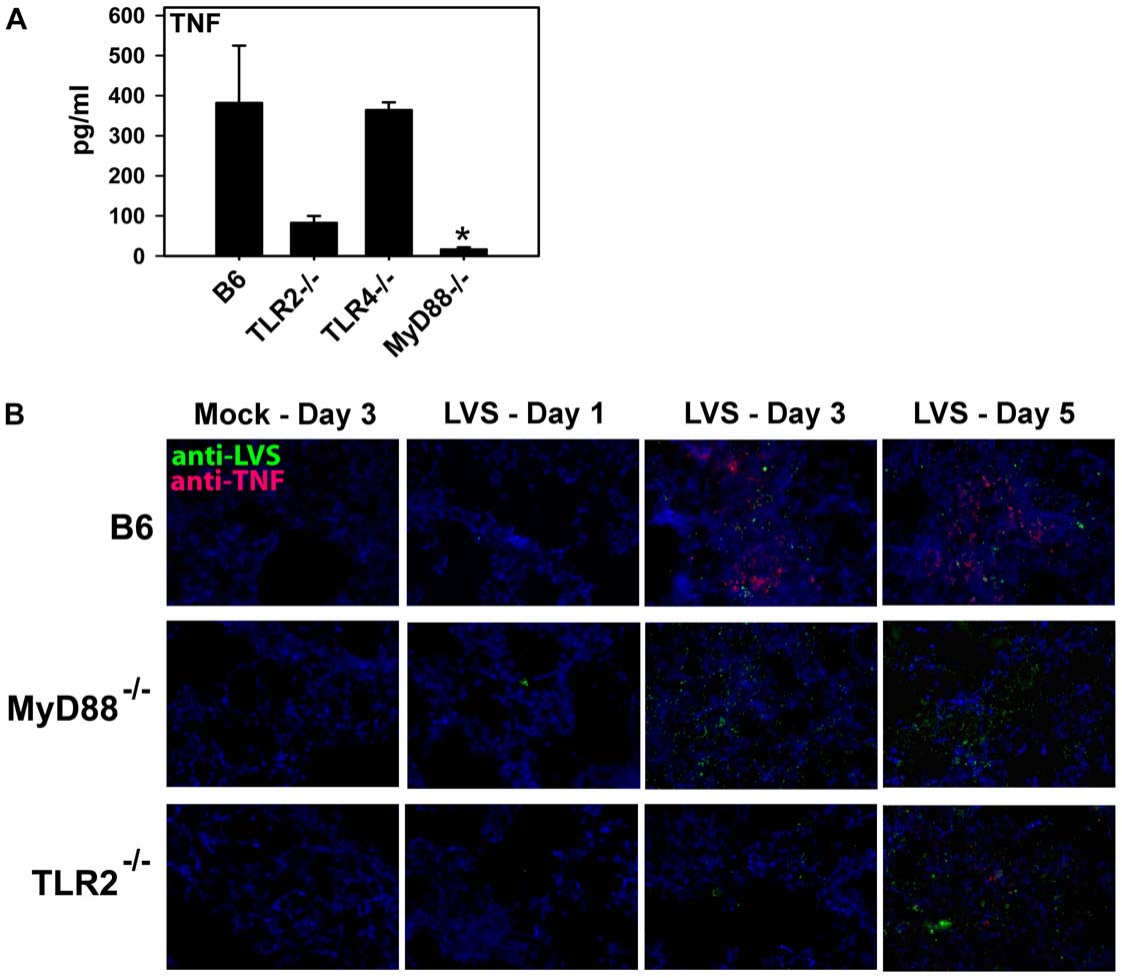
TNF expression in the lungs of mice infected i.n. with *F. tularensis* LVS. (A) Groups of 3–4 mice from the indicated strains were infected intranasally with 6,000 CFU of LVS. At day 5 post-infection, BALF was recovered from each mouse and cytokine levels quantified by BD™ Cytometric Bead Array (BD Biosciences, San Diego, CA). The data are expressed as the average cytokine level (+ SEM) from 3–4 individual mice. Significant differences among groups were determined by Kruskal-Wallis ANOVA on ranks followed by Dunn's post-hoc analysis; ^*^ *p*<0.05. (B) Groups of 2–3 B6, MyD88^−/−^ and TLR2^−/−^ mice were infected i.n. with 3,600 CFU of LVS or mock-infected and sacrificed after 1, 3, and 5 days post-infection. Lung tissue cryosections were prepared at the indicated time points and stained with purified goat anti-TNF (red) followed by Rhodamine Red-X-conjugated anti-goat Ig and analyzed by *in situ* immunofluorescence microscopy. Nuclei of cells (blue) were visualized via staining with 4′6′diamidino-2-phenylindole-dilactate (DAPI). The same sections were also stained for bacteria (green) with Alexa488-conjugated mouse anti-LVS LPS. Representative images from 2–3 mice are shown. Magnification for all images is 400×.

To assess the role of TLRs in the inflammatory cytokine response to *F. tularensis* infection *in vivo*, we analyzed proinflammatory cytokine expression in BAL fluid and performed *in situ* immunofluorescent staining for TNF expression in the lungs from mice infected intranasally with LVS. Analysis of BAL fluid demonstrated a significant reduction in secretion of TNF in the lungs of TLR2^−/−^ and MyD88^−/−^ mice compared to control mice at 5 days post-infection (Figure 4A). Similar results were obtained for IL-6 expression (data not shown). *In situ* TNF was clearly detectable by day 3 post-infection in the lungs of B6 mice (red stain in Figure 4B). No TNF expression could be detected in the lung tissue of MyD88^−/−^ mice, although bacteria were detectable by day 1 (Figure 4B). In lung tissue from TLR2^−/−^ mice, bacteria were detected as early as day 1, but TNF expression was just barely detectable by day 5 post-infection (Figure 4B). These results are consistent with the results of the *in vitro* experiments described above and demonstrate that inflammatory cytokine expression *in vivo* in the lungs in response to LVS infection is impaired in mice deficient in MyD88 or TLR2.

### TLR1^−/−^ and TLR6^−/−^ mice are not more susceptible to *F. tularensis* LVS infection than control mice

**Figure 5:**
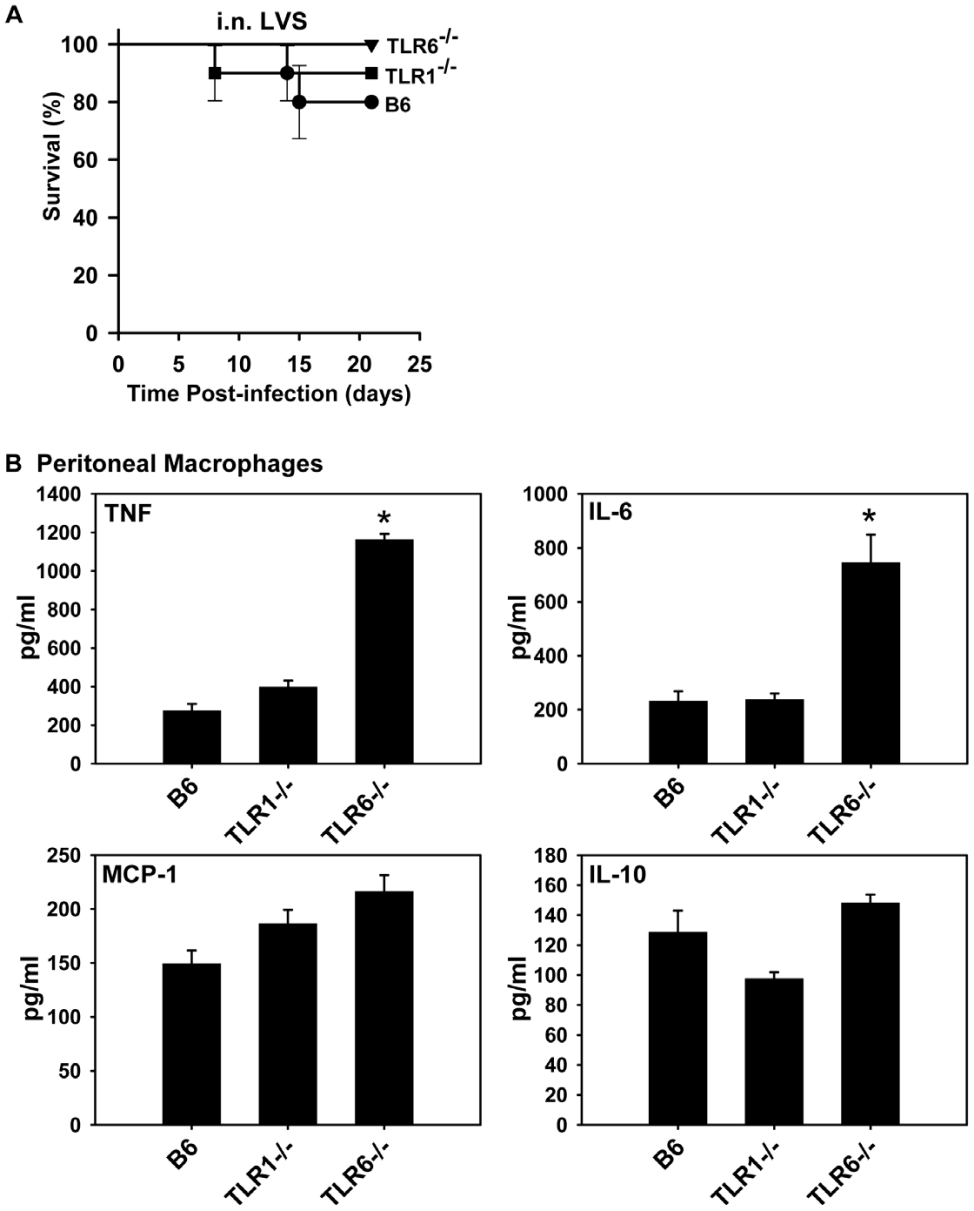
Survival rates and cytokine expression of TLR1^−/−^ and TLR6^−/−^ mice infected with *F. tularensis* LVS. (A) Survival curves are shown for intranasal infections with LVS. The data shown were pooled from two independent experiments with the i.n. inocula and group sizes as indicated: B6 (6,200 CFU, n = 10), TLR1^−/−^ (6,200 CFU, n = 10), TLR6^−/−^ (9,700 CFU, n = 10). (B) Proteose peptone-elicited peritoneal macrophages from B6, TLR1^−/−^, and TLR6^−/−^ mice were infected with LVS (MOI of 120), supernatants were collected from cultures at 4 h, and cytokines were quantified by BD™ Cytometric Bead Array (CBA) Mouse Inflammation Kit (BD Biosciences Pharmingen, San Diego, CA). The data are expressed as the average cytokine level (pg/ml) (+ SEM) in triplicate culture supernatants and are representative of 2–3 independent experiments. Significant differences among groups were determined by one-way ANOVA followed by Holm-Sidek post-hoc analysis; ^*^p<0.01. Cytokine and chemokine levels in supernatants from mock-infected cells were <20 pg/ml (data not shown).

TLR2 is known to pair with either TLR1 or TLR6 in the recognition of microbial ligands [27], [30], [45]. To investigate whether TLR1 or TLR6 might be required with TLR2 for a protective response to *F. tularensis*, survival experiments were performed in TLR1^−/−^ and TLR6^−/−^ mice (Figure 5A). The survival rate of these mice infected i.n. with LVS was indistinguishable from the survival rate of control mice indicating that neither TLR1 nor TLR6 is exclusively required for host resistance to LVS infection. Macrophages harvested from TLR1^−/−^ and TLR6^−/−^ mice expressed TNF, IL-6, MCP-1, and IL-10 in response to LVS infection *in vitro* at comparable or higher levels than B6 mice (Figure 5B). Thus, neither TLR1 nor TLR6 are exclusively required for *F. tularensis* recognition by TLR2.

## Discussion

The results of this study confirm and extend a recent study that demonstrated that TLR2 is critical for protection of mice against a primary pulmonary infection with *F. tularensis* LVS [32]. However, in contrast to a previous report [33], we show that the requirement for TLR2 is independent of the route of infection, since TLR2^−/−^ mice had significantly decreased survival rates compared to controls following infection by either the intranasal or the intradermal route. MyD88 also contributes significantly to survival of a primary pulmonary LVS infection, consistent with its reported role in the intradermal infection [33]. The results of this study further demonstrate that the host protective response to primary infection with *F. tularensis* subsp. *novicida* is also dependent on TLR2/MyD88 signaling. Importantly, TLR2^−/−^ macrophages are impaired in their ability to express pro-inflammatory cytokines and chemokines in response to *F. tularensis* LVS infection and pro-inflammatory cytokine expression is significantly impaired *in vivo* in the lungs of infected TLR2-deficient mice. Finally, this study revealed that neither TLR1^−/−^ nor TLR6^−/−^ mice were any more susceptible to LVS infection than control mice, although a previous study reported an exclusive role for TLR6 in the pro-inflammatory cytokine response of BM-derived dendritic cells to LVS *in vitro* [39].

As stated above, our conclusion that TLR2^−/−^ mice are more susceptible to primary infection with LVS regardless of the route of infection differs from conclusions drawn by Collazo *et al.* [33]. It should be noted, however, that the survival rate reported by Collazo *et al.* for i.d. LVS infection of TLR2^−/−^ mice (68%, n = 19) is not very different from the overall survival rate of 56% (n = 16) observed in this study, suggesting that the somewhat different results and the distinct conclusions may be due to differences in the infectious doses given, or simply due to different statistical treatment of the data. It is also important to point out that in our studies, as well as those of Collazo *et al.*, mice were infected intradermally with doses of LVS that are significantly below (1/40^th^ and 1/4^th^, respectively) the reported i.d. LD_50_ for B6 mice (∼2×10^6^ CFU) [21]. It seems very likely therefore that infection with higher doses of LVS would result in an even larger difference in survival rates between control mice and TLR2^−/−^ mice, as was observed by Malik *et al.* for intranasal LVS infections [32], and supporting the conclusion that TLR2 is important in both i.d. and i.n. infections. An accurate understanding of the role of particular TLRs in host responses to infections by different routes is important because of the implications for the rational design of vaccine-enhancing adjuvants.

Although MyD88^−/−^ and TLR2^−/−^ mice both demonstrated decreased survival rates compared to wild-type controls, the MyD88^−/−^ mice were significantly more susceptible to i.n. and i.d. LVS infection than were the TLR2^−/−^ mice. The reduced survival time for the MyD88^−/−^ mice also correlated with a greater increase in bacterial burden compared to TLR2^−/−^ mice beginning on day 3 post-infection. Notable also was the complete lack of detectable TNF in the lungs of the MyD88^−/−^ mice at days 1–5 post-infection. This is not surprising given the role of MyD88 as a critical signaling adaptor in numerous pro-inflammatory signaling pathways, including those activated via the IL-1β and IL-18 receptors, and in other TLR signaling pathways, *e.g.* TLR9 [46], [47]. Indeed, Mariathasan *et al.* recently demonstrated that *F. tularensis* subsp. *novicida* grew to higher titers in the organs of mice depleted of IL-1-β or IL-18 by treatment with neutralizing antibodies [25]. In that regard, however, Collazo *et al.* recently reported that survival of TLR9^−/−^, IL-1Rβ^−/−^ and IL-18^−/−^ mice after i.d. LVS infection was no different than for wild-type control mice [33]. Thus the basis for the difference in susceptibility to infection observed between MyD88^−/−^ and TLR2^−/−^ mice requires further study.

The survival studies reported herein indicate that neither TLR1 nor TLR6 is required exclusively to pair with TLR2 in recognition of *F. tularensis* LVS ligands. These results would appear inconsistent with a recent study that demonstrated a complete abrogation of TNF secretion by dendritic cells from TLR6-deficient mice but normal TNF secretion by TLR1-deficient cells [39]. We have also recently compared the TNF response to LVS by bone marrow-derived dendritic cells and have found no difference in the response between cells derived from TLR1^−/−^ or TLR6^−/−^ mice; however, the response from both were impaired relative to wild-type control dendritic cells (unpublished data). The reason for the discrepancy in results is not clear, but recently Re and colleagues have demonstrated that either TLR1 or TLR6 expressed in HEK-293 cells can recognize and mediate a response to LVS ligands [41] and the same group has recently identified specific ligands for TLR2/TLR1 in LVS [48]. Thus, the observation that deficiencies in either TLR1 or TLR6 have no impact on the survival of mice whereas TLR2 deficiency has a profound impact suggests that TLR1 and TLR6 may be redundant in the ability to recognize *F. tularensis* ligands in concert with TLR2. Interestingly, in our studies, macrophages from TLR6^−/−^ mice expressed significantly higher levels of TNF, IL-6, and MCP-1 than wild-type macrophages. It is not known if the absence of TLR6 leads to aberrant or increased activation through TLR2/TLR1 or if TLR6 normally functions to negatively regulate a signaling pathway.

Our studies and those of others [33], [49], [50] indicate that TLR4 plays no protective role in the host immune response to *F. tularensis* infection. This has been a somewhat surprising finding since *F. tularensis* is a Gram-negative bacterium and because a previous study reported that TLR4-defective mice (C3H/HeJ strain) were more susceptible to intradermal infection with LVS [51]. However, the LPS produced by *F. tularensis* has very little endotoxin activity compared to the LPS produced by *E. coli* or *Salmonella* species and has recently been shown to bind poorly, if at all, to TLR4 [52]–[54]. Moreover, other Gram-negative bacteria with atypical LPS have been reported to signal primarily through TLR2 rather than TLR4 [55]. It is interesting to note that the host inflammatory response to *F. tularensis* infection in the lungs of wild-type mice appears to be significantly delayed [2], [20], [56], suggesting the possibility that the absence of a potent TLR4 ligand in *F. tularensis* plays an important role in immune evasion. Indeed, recent studies have observed decreased virulence and enhanced innate immune responses for *F. tularensis* subsp. *novicida* mutants with altered lipid A moieties [57]. Although we have shown that TLR2^−/−^ mice are more susceptible to i.n. infection with *F. tularensis* subsp. *novicida*, a strain with a distinct, more biologically active LPS, and which is more highly virulent in mice than the LVS strain [10], TLR4 does not contribute significantly to host protection against this strain either.

TNF is well-known to play an important role in the immune response to *F. tularensis* infection and TNF-deficient mice succumb quickly to LVS infection, as do IL-12-deficient and IFN-γ-deficient mice [58]–[63]. Such studies support a model in which early expression of TNF, IL-12 and IFN-γ by diverse myeloid and lymphoid cell types in response to *F. tularensis* infection induces recruitment of inflammatory cells and IFN-γ production primarily from NK cells and dendritic cells [64] that in turn further activate macrophages and dendritic cells and induce Th1 immunity. The significant reduction in the expression of TNF by TLR2-deficient macrophages infected *in vitro* and the significant reduction of TNF expression in the lungs of TLR2^−/−^ mice infected with LVS is striking and suggests that the increased susceptibility of TLR2-deficient mice to infection can perhaps be accounted for solely by the impairment of TNF expression *in vivo* early in the infection. However, other TLR2-dependent pro-inflammatory cytokines and chemokines may also play important roles in the protective host immune response as illustrated by the TLR2-dependent induction of IL-6 and MCP-1 in infected macrophages. Although our data do not reveal which cells harbor LVS in the lungs or which cells are producing proinflammatory cytokines, others have shown that alveolar macrophages, dendritic cells, and neutrophils are the primary cells in the lung that are infected with *F. tularensis* [2], [65]. Future studies will attempt to delineate the role of the expression of individual cytokines by specific cell types in the TLR-dependent host response to *F. tularensis* infection.

Finally, the components of *F. tularensis* that are responsible for activating innate immune responses in the host are just beginning to be identified [31], [66]. TLR2 is expressed on many cell types and has been reported to bind to a broad array of microbial components [67], most notably lipoproteins [68], but also peptidoglycan [35], [69], and recently a bacterial porin [70]. TLR2 has also been shown to play a role in the host response to a number of infections by both Gram-positive and Gram-negative bacteria, including *Staphylococcus aureus* [71], *Streptococcus pneumonia* [72], *Legionella pneumophila* [73], [74], and *Porphyromonas gingivalis* [75]; it also plays an important role in the responses to a number of bacteria that express an atypical LPS [55]. Identification of the TLR2 ligands responsible for activating host protective responses to *F. tularensis*, as well as other possible *F. tularensis* PAMPs, will be important for a complete understanding of *F. tularensis* pathogenesis and therefore an important goal of future studies.
